# High-throughput multiplex detection of respiratory pathogens: multicenter evaluation of the NxTAG Respiratory Pathogen Panel v2 assay

**DOI:** 10.1128/jcm.01743-25

**Published:** 2026-03-19

**Authors:** Marisa C. Nielsen, Ping Ren, Sri Bharathi Kavuri, Karissa Culbreath, Rangaraj Selvarangan, Dithi Banerjee, Blake W. Buchan, Rachel Behounek, Janet Farhang

**Affiliations:** 1 Pathology and Laboratory Medicine, Boston Medical Center and Boston University Chobanian & Avedisian School of Medicine 12259, Boston, Massachusetts, USA; 2 Department of Pathology, University of Texas Medical Branch 198642, Galveston, Texas, USA; 3 TriCore Reference Laboratories 159777 , Albuquerque, New Mexico, USA; 4 Children's Mercy Kansas City 4204 , Kansas City, Missouri, USA; 5 Medical College of Wisconsin 5506, Milwaukee, Wisconsin, USA; 6 Luminex Corporation 17737 , Austin, Texas, USA; Cleveland Clinic, Cleveland, Ohio, USA

**Keywords:** respiratory tract infections, multiplex PCR, molecular diagnostics, syndromic testing

## Abstract

Respiratory tract infections remain a major cause of global morbidity, highlighting the need for rapid and accurate diagnostic testing. This multicenter study evaluated the clinical and analytical performance of the updated NxTAG Respiratory Pathogen Panel v2 (RPPv2), a high-throughput multiplex molecular assay designed to detect 21 viral and bacterial respiratory pathogens. A total of 2,145 nasopharyngeal swab specimens, including both prospectively enrolled and archived specimens, were analyzed across five U.S. clinical sites. The NxTAG RPPv2 was assessed against FDA-cleared molecular assays and bi-directional sequencing, with contrived specimens used to supplement low-prevalence targets. Valid results were obtained for 99.7% of specimens after a single retest, with minimal performance differences between fresh and frozen specimens. Among viral targets, 15 of 18 (one viral target did not have clinical positive specimens for analysis) achieved ≥95% positive percent agreement (PPA) across the prospective and pre-selected arms. For bacterial targets, *Mycoplasma pneumoniae* and *Chlamydia pneumoniae* showed PPA values of 92.3% and 100%, respectively. Lower PPA values were observed for parainfluenza 4 (90.3%). Negative percent agreement (NPA) was consistently high across targets, with all viral and bacterial analytes achieving ≥99.2%. Reproducibility across sites and operators was 99.9%. Overall, the NxTAG RPPv2 demonstrated high accuracy, reproducibility, and operational efficiency, supporting its clinical utility for rapid and reliable diagnosis of respiratory tract infections.

**IMPORTANCE:**

Respiratory infections remain a major global health challenge, particularly during seasonal epidemics and pandemics. Rapid and accurate pathogen detection is essential to guide treatment decisions, limit transmission, and reduce unnecessary antibiotic use. This multicenter study evaluated the updated NxTAG Respiratory Pathogen Panel v2 (RPPv2), a high-throughput molecular assay capable of detecting 22 common viral and bacterial respiratory pathogens from a single sample. The panel demonstrated high accuracy, reproducibility, and reliability across multiple clinical sites and sample types, including both fresh and frozen specimens. These findings support the NxTAG RPPv2 as a comprehensive, efficient diagnostic tool that can support respiratory infection diagnosis and contribute to multifaceted antimicrobial stewardship efforts.

## INTRODUCTION

Respiratory tract infections (RTIs) are among the most frequent causes of outpatient visits, hospitalizations, and antimicrobial prescriptions worldwide, contributing significantly to morbidity, mortality, and healthcare costs (1). The clinical manifestations of RTIs often overlap among viral and bacterial etiologies, making accurate diagnosis challenging (2).

The introduction of molecular diagnostics has transformed respiratory pathogen testing. Over the past two decades, polymerase chain reaction (PCR) based technologies, particularly real-time and multiplex PCR platforms, have enabled simultaneous detection of multiple viral and bacterial agents in a single assay. These platforms have improved diagnostic yield in syndromic presentations where clinical features alone are insufficient to distinguish between viral and bacterial etiologies (3, 4). Multiplex respiratory panels (MRPs) have become integral to clinical practice, particularly during periods of high viral circulation, such as influenza seasons and the COVID-19 pandemic. Their adoption has demonstrated value in reducing diagnostic uncertainty, minimizing unnecessary antimicrobial use, optimizing patient isolation, and strengthening infection control measures (5, 6). Commercially available MRPs, including FDA-cleared systems, such as the BioFire FilmArray and GenMark ePlex, offer laboratories diverse options that vary in throughput, automation, and pathogen coverage.

The NxTAG Respiratory Pathogen Panel (NxTAG RPP, Luminex) combines bead-based hybridization with a closed-tube, high-throughput workflow on the MAGPIX system, enabling efficient detection of respiratory pathogens in larger testing volumes (7). The updated NxTAG RPPv2 expands pathogen coverage and incorporates workflow refinements aimed at enhancing diagnostic accuracy and laboratory efficiency.

This multicenter study provides the first analytical evaluation of the NxTAG RPPv2 for the detection of respiratory pathogens across multiple U.S. laboratories. Performance characteristics were assessed in comparison with established molecular reference methods and existing multiplex platforms, with emphasis on analytical accuracy, reproducibility, and operational workflow. Findings from this study aim to inform laboratories, clinicians, and public health stakeholders of the potential clinical utility of this updated assay in improving the diagnosis and management of RTIs.

## MATERIALS AND METHODS

### NxTAG RPPv2 testing workflow

For NxTAG RPPv2 testing, 10 µL of MS2 internal control was added to easyMAG or EMAG disposable cartridges together with 200 µL of patient specimen. Nucleic acids were extracted with a final elution volume of 110 µL. Thirty-five microliters of specimen or control eluate was transferred to the NxTAG RPPv2 assay plate, which was placed in a preheated thermal cycler for PCR amplification. Upon completion of thermal cycling, the assay plate was analyzed using the Luminex MAGPIX instrument. Detailed extraction procedures and thermal cycling parameters are described in the NxTAG RPPv2 Instructions for Use (https://www.accessdata.fda.gov/cdrh_docs/pdf23/K231758.pdf).

### Bidirectional sequencing

Briefly, two independent PCR assays, followed by sequencing assays, were designed and validated in-house at Luminex for each analyte. Whenever feasible, PCR primers used for bidirectional sequencing (BDS) targeted genomic regions distinct from those targeted by the candidate device for the corresponding analyte. Confirmation of fragment analysis-positive results was performed using Sanger (dideoxy or chain termination) sequencing on an ABI 3730xl DNA Analyzer.

Sequencing data from both forward and reverse directions were evaluated. Acceptable sequence quality required at least 100 contiguous bases meeting the following criteria: (i) ≥90% of bases with a PHRED quality score ≥20 (base-call accuracy ≥99%); (ii) ≤5% ambiguous bases (“N”s); (iii) an expected value (E-value) ≤1 × 10^−30^ when compared with the reference sequence; and (iv) BLAST analysis demonstrating ≥95% query coverage and ≥95% nucleotide identity relative to the reference sequence.

A specimen was classified as negative if both BDS assays were negative. A specimen was classified as positive for a given analyte if both sequencing directions from at least one bidirectional sequencing assay met or exceeded the predefined quality and alignment criteria. BDS was used to differentiate influenza A H1 from influenza A 2009 H1N1, as this distinction is not reported by the NxTAG RPP assay. In addition, BDS was used to confirm the positivity of archived specimens selected based on standard-of-care (SoC) results, as these specimens had been stored frozen for extended periods.

### Study design

A multisite clinical study was conducted to evaluate the performance of the NxTAG RPPv2 assay for the detection of nucleic acids from respiratory viruses and bacteria. Nasopharyngeal swab (NPS) specimens were prospectively enrolled between October 2022 and April 2023 at five geographically diverse clinical sites across the U.S. NxTAG RPPv2 testing was performed at three of the five sites by trained personnel using the Luminex MAGPIX system. Nucleic acid extraction was conducted on the bioMérieux eMAG and easyMAG platforms (Durham, NC).

Eligible specimens met all of the following criteria: (i) collected and preserved in 3 mL of Universal/Viral Transport Media; (ii) obtained from male or female subjects of any age presenting with clinical signs and symptoms of RTI, whether hospitalized, in the emergency department, attending an outpatient clinic, or residing in a long-term care facility; (iii) accompanied by a valid requisition; (iv) containing a minimum residual volume of ≥1.35 mL; and (v) received in good condition without evidence of leakage or drying.

To supplement the prospective enrollment, 320 archived clinical specimens were included based on SoC testing results and confirmed by BDS prior to inclusion. Archived specimens were collected between January 2020 and May 2022. Specimens were stored frozen at −80°C and shipped on dry ice to the testing sites. Archived positive and negative specimens were randomized, blinded, and tested at four participating sites.

Due to the low prevalence of prospective and archived clinical specimens positive for *Chlamydia pneumoniae*, respiratory syncytial virus B (RSV B), pre-2009 pandemic influenza A H1N1, and coronavirus 229E, contrived specimens were generated to assess assay performance. Negative NPS specimens were spiked with representative strains at 2×, 10×, and 100× the assay’s limit of detection (LoD), except for influenza A H1N1, which was tested at 2× and 10× LoD using vendor-supplied stock concentrations (Zeptometrix Corporation, Buffalo, NY). Contrived specimens were randomized, blinded, and tested alongside archived specimens at two testing sites between December 2021 and January 2022. Results from contrived specimens were analyzed separately from clinical specimen data.

For reproducibility assessment, a panel consisting of one negative specimen and four multi-analyte (MA) specimens was prepared in a negative matrix at two concentrations: low positive (1.5–3× LoD) and moderate positive (5–9× LoD).

### Statistical analysis

Positive percent agreement (PPA) and negative percent agreement (NPA) of the NxTAG RRPv2 assay were calculated relative to comparator methods, including NxTAG RPP, the DiaSorin MDx Simplexa COVID-19 Direct Kit for SARS-CoV-2, and BDS when results were discrepant between NxTAG RPPv2 and NxTAG RPP. In addition, PPA and NPA of NxTAG RRPv2 relative to SoC results provided by participating laboratories were also evaluated. For comparisons against SoC results, discrepant findings were adjudicated using NxTAG RPP and BDS. Test concordance was classified as true positive (TP), true negative (TN), false positive (FP), or false negative (FN). Results were expressed as proportions with two-sided 95% confidence intervals (CIs) according to the Wilson score method.

All statistical analyses were performed using SAS software, version 9.4_M4 (SAS Institute, Cary, NC), operating on an Oracle Linux 6.8 (64-bit) system.

## RESULTS

### Demographics, specimens, and invalid results

**Fig 1:**
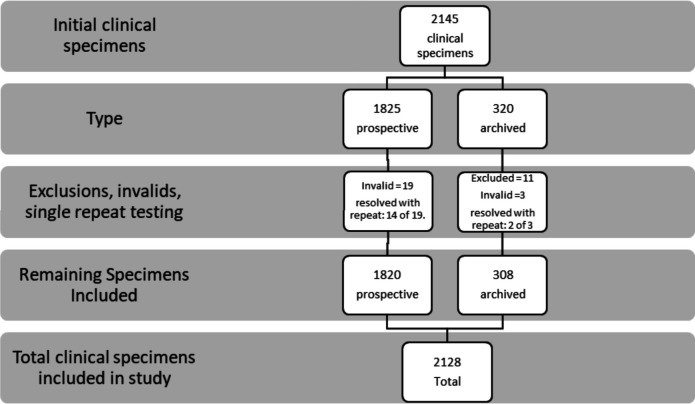
Flow diagram of clinical specimen enrollment, exclusions, and final inclusion.

A total of 2,145 clinical specimens were included in the study, comprising 1,825 prospectively collected and 320 archived specimens. Valid NxTAG RPPv2 results were obtained for 2,123 (98.97%) specimens on the first attempt and for 2,139 (99.72%) specimens after a single retest resolved 16 of the 22 initially invalid results (Fig. 1).

Among the 1,825 prospective specimens, 19 (1.0%) initially yielded invalid results with the NxTAG RPPv2 assay; repeat testing resolved 14 of these, resulting in 1,820 (99.7%) evaluable specimens for analysis (Fig. 1). Of the 320 archived specimens, 11 were excluded due to insufficient residual volume for confirmatory testing. Three initially invalid results were observed in this group, two of which were successfully resolved upon repeat testing, leaving 308 archived specimens included in the final data set (Fig. 1).

Demographic characteristics of evaluable specimens are summarized in Table 1. Among prospective specimens (*n* = 1,820), 55.1% were from female and 44.9% from male patients, with most patients aged 21–65 years (35.4%) or under 21 years (50.6%). In the archived group (*n* = 308), 45.8% were female and 48.4% male, with a higher proportion of pediatric and young adult specimens (25.6% aged 0–1 year, 15.9% aged >1–5 years, and 29.5% aged >5–21 years). The majority of prospective specimens were collected from emergency department patients (63.0%), followed by outpatients (19.6%) and hospitalized individuals (16.7%). In contrast, most archived specimens (91.6%) had unknown clinical status due to specimen banking and limited associated metadata.

**TABLE 1:** Demographic details for clinical specimens

	Prospective (*N* = 1,820)	Archived (*N* = 308)
	# Specimens (%)	# Specimens (%)
Gender		
Male	817 (44.9%)	149 (48.4%)
Female	1,003 (55.1%)	141 (45.8%)
Unknown	0 (0.0%)	18 (5.8%)
**Total**	1,820 (100.0%)	308 (100.0%)
Age (years)		
0–1	337 (18.5%)	79 (25.6%)
>1–5	253 (13.9%)	49 (15.9%)
>5–21	331 (18.2%)	91 (29.5%)
>21–65	645 (35.4%)	42 (13.6%)
>65	242 (13.3%)	16 (5.2%)
Unknown	12 (0.7%)	31 (10.1%)
**Total**	1,820 (100.0%)	308 (100.0%)
Subject status		
Outpatient	356 (19.6%)	11 (3.6%)
Hospitalized	304 (16.7%)	11 (3.6%)
Emergency room	1,146 (63.0%)	4 (1.3%)
Unknown	14 (0.8%)	282 (91.6%)
**Total**	1,820 (100.0%)	308 (100.0%)

### Extraction platform comparison

Both the easyMAG and eMAG extraction platforms were used in this clinical study. A total of 2,344 unique specimens were tested, of which 1,277 were extracted using the easyMAG platform, and 1,067 were extracted using the eMAG platform. No significant differences in clinical performance were observed between specimens processed on the easyMAG and eMAG platforms. Equivalency between the two extraction platforms was further supported by a LoD comparison study conducted during the development of the NxTAG RPPv2 assay (data not shown).

### Accuracy

The clinical performance of each NxTAG RPPv2 target was evaluated against an FDA-cleared molecular assay and/or a composite reference method consisting of PCR, followed by bidirectional sequencing. For each target on the panel (Table S1), PPA, NPA, and corresponding 95% CIs were calculated (Table 2).

**TABLE 2:** NxTAG RPPv2 performance for the prospective clinical specimens

Pathogen target		Positive percent agreement			Negative percent agreement		
Prospective		TP^*a*^/(TP+FN^*a*^)	PPA^*a*^ (%)	95% CI^*a*^ (%)	TN^*a*^/(TN+FP^*a*^)	NPA^*a*^ (%)	95% CI (%)
Viruses							
Adenovirus	Fresh	39/39	100	91.0–100	733/736	99.6	98.8–99.9
	Frozen	55/59	93.2	83.8–97.3	976/980	99.6	99.0–99.8
	**Combined**	**94/98** ^*b*^	**95.9**	**90.0–98.4**	**1,709/1,716** ^*c*^	**99.6**	**99.2–99.8**
Coronavirus 229E	Fresh	2/2	100	34.2–100	773/773	100	99.5–100
	Frozen	5/5	100	56.6–100	1,033/1,034	99.9	99.5–100
	**Combined**	**7/7**	**100**	**64.6–100**	**1,806/1,807**	**99.9**	**99.5–100**
Coronavirus HKU1	Fresh	8/8	100	67.6–100	767/767	100	99.5–100
	Frozen	12/13	92.3	66.7–98.6	1,026/1,026	100	99.6–100
	**Combined**	**20/21** ^*d*^	**95.2**	**77.3–99.2**	**1,793/1,793**	**100**	**99.8–100**
Coronavirus NL63	Fresh	25/27	92.6	76.6–97.9	748/748	100	99.5–100
	Frozen	23/25	92.0	75.0–97.8	1,014/1,014	100	99.6–100
	**Combined**	**48/52**	**92.3**	**81.8–97.0**	**1,762/1,762**	**100**	**99.8–100**
Coronavirus OC43	Fresh	10/10	100	72.2–100	765/765	100	99.5–100
	Frozen	29/29	100	88.3–100	1,010/1,010	100	99.6–100
	**Combined**	**39/39**	**100**	**91.0–100**	**1,775/1,775**	**100**	**99.8–100**
Human metapneumovirus	Fresh	87/87	100	95.8–100	680/688	98.8	97.7–99.4
	Frozen	70/70	100	94.8–100	963/969	99.4	98.7–99.7
	**Combined**	**157/157**	**100**	**97.6–100**	**1,643/1,657** ^*e*^	**99.2**	**98.6–99.5**
Influenza A	Fresh	20/20	100	83.9–100	753/755	99.7	99.0–99.9
	Frozen	54/54	100	93.4–100	984/985	99.9	99.4–100
	**Combined**	**74/74**	**100**	**95.1–100**	**1,737/1,740** ^*f*^	**99.8**	**99.5–99.9**
Influenza A H1pdm09	Fresh	9/9	100	70.1–100	765/765	100	99.5–100
	Frozen	22/22	100	85.1–100	1,017/1,017	100	99.6–100
	**Combined**	**31/31**	**100**	**89.0–100**	**1,782/1,782**	**100**	**99.8–100**
Influenza A H1	Fresh	0/0	N/A^*a*^	N/A	774/774	100	99.5–100
	Frozen	0/0	N/A	N/A	1,039/1,039	100	99.6–100
	**Combined**	**0/0**	**N/A**	**N/A**	**1,813/1,813**	**100**	**99.8–100**
Influenza A H3	Fresh	11/11	100	74.1–100	764/764	100	99.5–100
	Frozen	34/36	94.4	81.9–98.5	1,002/1,003	99.9	99.4–100
	**Combined**	**45/47** ^*g*^	**95.7**	**85.8–98.8**	**1,766/1,767**	**99.9**	**99.7–100**
Influenza B	Fresh	5/5	100	56.6–100	770/770	100	99.5–100
	Frozen	6/6	100	61.0–100	1,033/1,033	100	99.6–100
	**Combined**	**11/11**	**100**	**74.1–100**	**1,803/1,803**	**100**	**99.8–100**
Parainfluenza 1	Fresh	7/7	100	64.6–100	768/768	100	99.5–100
	Frozen	11/11	100	74.1–100	1,028/1,028	100	99.6–100
	**Combined**	**18/18**	**100**	**82.4–100**	**1,796/1,796**	**100**	**99.8–100**
Parainfluenza 2	Fresh	4/5	80.0	37.6–96.4	770/770	100	99.5–100
	Frozen	5/5	100	56.6–100	1,034/1,034	100	99.6–100
	**Combined**	**9/10**	**90.0**	**59.6–98.2**	**1,804/1,804**	**100**	**99.8–100**
Parainfluenza 3	Fresh	19/19	100	83.2–100	756/756	100	99.5–100
	Frozen	23/23	100	85.7–100	1,015/1,016	99.9	99.4–100
	**Combined**	**42/42**	**100**	**91.6–100**	**1,771/1,772**	**99.9**	**99.7–100**
Parainfluenza 4	Fresh	2/3	66.7	20.8–93.9	770/772	99.7	99.1–99.9
	Frozen	11/12	91.7	64.6–98.5	1,026/1,027	99.9	99.5–100
	**Combined**	**13/15^*h*^**	**86.7**	**62.1–96.3**	**1,796/1,799^*i*^**	**99.8**	**99.5–99.9**
RSV A	Fresh	10/10	100	72.2–100	764/765	99.9	99.3–100
	Frozen	45/45	100	92.1–100	992/994	99.8%	99.3–99.9
	**Combined**	**55/55**	**100**	**93.5–100**	**1,756/1,759^*j*^**	**99.8**	**99.5–99.9**
RSV B	Fresh	3/3	100	43.9–100	772/772	100	99.5–100
	Frozen	17/17	100	81.6–100	1,022/1,022	100	99.6–100
	**Combined**	**20/20**	**100**	**83.9–100**	**1,794/1,794**	**100**	**99.8–100**
Rhinovirus/enterovirus	Fresh	123/132	93.2	87.5–96.4	643/643	100	99.4–100
	Frozen	228/237	96.2	92.9–98.0	801/802	99.9	99.3–100
	**Combined**	**351/369^*k*^**	**95.1**	**92.4–96.9**	**1,444/1,445**	**99.9**	**99.6–100**
SARS-CoV-2	Fresh	103/106	97.2	92.0–99.0	656/660	99.4	98.5–99.8
	Frozen	126/128	98.4	94.5–99.6	902/909	99.2	98.4–99.6
	**Combined**	**229/234^*l*^**	**97.9**	**95.1–99.1**	**1,558/1,569^*m*^**	**99.3**	**98.7–99.6**
Bacteria							
*Chlamydia pneumoniae*	Fresh	0/0	N/A	N/A	775/775	100	99.5–100
	Frozen	0/0	N/A	N/A	1,039/1,039	100	99.6–100
	**Combined**	**0/0**	**N/A**	**N/A**	**1,814/1,814**	**100**	**99.8–100**
*Mycoplasma pneumoniae*	Fresh	0/0	N/A	N/A	775/775	100	99.5–100
	Frozen	0/0	N/A	N/A	1,039/1,039	100	99.8–100
	**Combined**	**0/0**	**N/A**	**N/A**	**1,814/1,814**	**100**	**99.8–100**

^*a*^ TP, true positive; TN, true negative; FP, false positive; FN, false negative; PPA, positive percentage agreement; NPA, negative percentage agreement; CI, confidence interval; N/A, not applicable.

^*b*^ Two of the four adenovirus false negatives were negative by bidirectional sequencing, and two were not tested due to volume limitations.

^*c*^ Four of the seven adenovirus false positives were positive by molecular standard-of-care testing performed at the participating laboratories.

^*d*^ One coronavirus HKU1 false negative was negative by molecular standard-of-care testing performed at the participating laboratory.

^*e*^ Seven of the 14 human metapneumovirus false positives were not tested by bidirectional sequencing due to volume limitations. Two of the 14 were positive by molecular standard-of-care testing performed at the participating laboratory.

^*f*^ Three influenza A false positives were positive by bidirectional sequencing.

^*g*^ One of the two influenza A H3 false negatives was negative by molecular standard-of-care testing performed at the participating laboratory.

^*h*^ One of the two parainfluenza 4 false negatives was negative by molecular standard-of-care testing performed at the participating laboratory.

^*i*^ Two of the three parainfluenza four false positives were positive by molecular standard-of-care testing performed at the participating laboratories.

^*j*^ One of the three RSV A false positives was positive by molecular standard-of-care testing performed at the participating laboratory.

^*k*^ Eight of the 18 rhinovirus/enterovirus false negatives were negative by bidirectional sequencing, and an additional three false negatives were negative by molecular standard-of-care testing performed at the participating laboratories. Five were not tested due to volume limitations.

^*l*^ Two of the five SARS-CoV-2 false negatives were negative by molecular standard-of-care testing performed at the participating laboratories.

^*m*^ Six of the eleven SARS-CoV-2 false positives were positive by molecular standard-of-care testing performed at the participating laboratories.

#### Prospective specimens

The assay demonstrated accurate diagnostic performance across a broad range of respiratory pathogens in both fresh and frozen specimens. Among viral targets, 15 of 18 achieved a PPA of ≥95% (no clinical positive specimens for influenza A H1 were obtained). High concordance was observed for adenovirus (95.9% PPA, 99.6% NPA), influenza A/B/H1pdm09 (100% PPA, NPA), coronaviruses OC43 (100% PPA, NPA) and 229E (100% PPA, 99.9% NPA), rhinovirus/enterovirus (95.1% PPA, 99.9% NPA), and RSV A/B (100% PPA and NPA 99.8% for RSV A and 100% for RSV B, respectively). Lower PPA values were noted for coronavirus HKU1 (94.3%), coronavirus NL63 (92.3%), and parainfluenza 4 (90.3%). SARS-CoV-2 demonstrated 97.9% PPA and 99.3% NPA.

For bacterial targets, *Chlamydia pneumoniae* and *Mycoplasma pneumoniae* showed 100% NPA, though no positive clinical detections were available. Overall, all viral targets and both bacterial targets achieved ≥99.2% NPA, with minimal performance differences observed between fresh and frozen specimens.

The NxTAG RPPv2 detected multiple pathogens in 125 prospective specimens, representing 11.3% (125/1,103) of positive prospective specimens and 6.9% (125/1,820) of all prospective specimens included in the performance analysis. Most co-infections (116/125, 92.8%) involved two pathogens, while 7.2% (9/125) involved three pathogens. Of these, 84.8% (106/125) were concordant with the comparator methods (Table S2).

#### Archived specimens

Similarly, the NxTAG RPPv2 assay demonstrated high accuracy in the archived specimen group (Table 3). For most viral and bacterial targets with sufficient positive specimens, PPA was high, often 100%, with consistently strong NPA (typically ≥99%). Notably, adenovirus, coronavirus 229E, influenza A, influenza B, parainfluenza 1 and 2, and *Chlamydia pneumoniae* all showed perfect or near-perfect concordance. Slightly lower PPA values were observed for coronavirus HKU1, influenza A H1pdm09, parainfluenza 4, and *Mycoplasma pneumoniae* (ranging 92% to 97%), while maintaining high NPA. For targets where prospective enrollment provided sufficient statistical power (*N* ≥ 50 clinical comparator method positives for influenza A and SARS-CoV-2, *N* ≥ 30 clinical comparator method positives for all others), no archived positive specimens were enrolled; however, NPA remained near 100% across these analytes.

**TABLE 3:** NxTAG RPPv2 performance for the archived clinical specimens

Pathogen target	Positive percent agreement			Negative percent agreement		
	TP^*a*^/(TP+FN^*a*^)	PPA^*a*^ (%)	95% CI^*a*^ (%)	TN^*a*^/(TN+FP^*a*^)	NPA^*a*^ (%)	95% CI (%)
Viruses						
Adenovirus	1/1	100	20.7–100	307/307	100	98.8–100
Coronavirus 229E	11/11	100	74.1–100	297/297	100	98.7–100
Coronavirus HKU1	30/32	93.8	79.9–98.3	276/276	100	98.6–100
Coronavirus NL63	0/0	N/A	N/A	308/308	100	98.8–100
Coronavirus OC43	0/0	N/A	N/A	308/308	100	98.8–100
Human metapneumovirus	0/0	N/A	N/A	308/308	100	98.8–100
Influenza A	30/30	100	88.6–100	277/278	99.6	98.0–99.9
Influenza A H1pdm09	29/30	96.7	83.3–99.4	278/278	100	98.6–100
Influenza A H1	0/0	N/A	N/A	277/278	99.6	98.0–99.9
Influenza A H3	0/0	N/A	N/A	278/278	100	98.6–100
Influenza B	30/30	100	88.6–100	278/278	100	98.6–100
Parainfluenza 1	29/29	100	88.3–100	279/279	100	98.6–100
Parainfluenza 2	30/30	100	88.6–100	278/278	100	98.6–100
Parainfluenza 3	0/0	N/A	N/A	307/307	100	98.8–100
Parainfluenza 4	15/16	93.8	71.7–98.9	292/292	100	98.7–100
RSV A	0/0	N/A	N/A	305/307	99.3	97.7–99.8
RSV B	0/0	N/A	N/A	306/307	99.7	98.2–99.9
Rhinovirus/enterovirus	1/1	100	20.7–100	302/306	98.7	96.7–99.5
SARS-CoV-2	0/0	N/A	N/A	0/0	N/A	N/A
Bacteria						
*Chlamydia pneumoniae*	14/14	100	78.5–100	293/294	99.7	98.1–99.9
*Mycoplasma pneumoniae*	48/52	92.3	81.8–97.0	256/256	100	98.5–100

^*a*^ TP, true positive; TN, true negative; FP, false positive; FN, false negative; PPA, positive percentage agreement; NPA, negative percentage agreement; CI, confidence interval; N/A, not applicable.

#### Contrived specimens

Contrived specimens were generated to supplement low-prevalence targets not adequately represented in the clinical data set, and results were analyzed separately. The combined PPA across analytes ranged from 98% for RSV B and *C. pneumoniae* to 100% for all other targets, while NPA remained consistently at or near 100% across all contrived specimens (Table 4).

**TABLE 4:** NxTAG RPPv2 performance for the contrived specimens

Pathogen target		Positive percent agreement			Negative percent agreement		
		TP^*a*^/(TP+FN^*a*^)	PPA^*a*^ (%)	95% CI^*a*^ (%)	TN^*a*^/(TN+FP^*a*^)	NPA^*a*^ (%)	95% CI (%)
**Analyte**	**LoD** ^***a***^	**Viruses**					
Coronavirus 229E	2×	25/25	100	86.7–100	76/76	100	95.2–100
	10×	12/12	100	75.8–100	50/50	100	92.9–100
	100×	12/12	100	75.8–100	24/24	100	86.2–100
	**Combined**	**49/49**	**100**	**92.7–100**	**150/150**	**100**	**97.5–100**
Influenza A (matrix)	2×	26/26	100	87.1–100	75/75	100	95.1–100
	10×	24/24	100	86.2–100	38/38	100	90.8–100
	100×	0/0	N/A	N/A	36/36	100	90.4–100
	**Combined**	**50/50**	**100**	**92.9–100**	**149/149**	**100**	**97.5–100**
Influenza A H1 (subtype)	2×	26/26	100	87.1–100	75/75	100	95.1–100
	10×	24/24	100	86–100	38/38	100	90.8–100
	100×	0/0	N/A	N/A	36/36	100	90.4–100
	**Combined**	**50/50**	**100**	**92.9–100**	**149/149**	**100**	**97.5–100**
RSV B	2×	24/25	96.0	80.5–99.3	76/76	100	95.2–100
	10×	13/13	100	77.2–100	48/49	98.0	89.3–99.6
	100×	12/12	100	75.8–100	24/24	100	86.2–100
	**Combined**	**49/50**	**98.0**	**89.5–99.6**	**148/149**	**99.3**	**96.3–99.9**
**Analyte**	**LoD**	**Bacteria**					
*Chlamydia pneumoniae*	2×	25/25	100	86.7–100	76/76	100	95.2–100
	10×	12/13	92.3	66.7–98.6	49/49	100	92.7–100
	100×	12/12	100	75.8–100	24/24	100	86.2–100
	**Combined**	**49/50**	**98.0**	**89.5–99.6**	**149/149**	**100**	**97.5–100**

^*a*^ TP, true positive; TN, true negative; FP, false positive; FN, false negative; PPA, positive percentage agreement; NPA, negative percentage agreement; CI, confidence interval; N/A, not applicable; LoD, limit of detection.

### Comparison with SoC testing

The NxTAG RPPv2 was also compared with SoC molecular assays used by participating laboratories for prospective and archived specimens. Table 5 summarizes the distribution of SoC assays performed across multiple diagnostic platforms, encompassing 12 manufacturers and 21 distinct assays. The BioFire Respiratory Panel 2.1 accounted for the largest number of tests (*n* = 694), followed by the NeuMoDx 96 SARS-CoV-2 assay (*n* = 396).

**TABLE 5:** Standard of care instrumentation and assays

Instrument and assay	Number of tests
Abbott RealTime SARS-CoV-2	125
Abbott RESP-4-PLEX assay	118
BioFire Respiratory Panel 2.1	700
BioFire Respiratory Panel 2.0	117
BioFire Respiratory Panel 1.7	42
NeuMoDx 96 Molecular System SARS-CoV-2	398
Cepheid Xpert Xpress SARS-CoV-2/Flu/RSV	104
Cepheid Xpert Xpress Flu/RSV	26
Cepheid Xpert Xpress SARS-CoV-2	10
Cepheid Xpert Xpress SARS-CoV-2 plus	50
Cepheid Xpert Xpress SARS-CoV-2/Flu	63
Cobas SARS-CoV-2 Flu A/B	13
DiaSorin MDx Simplexa COVID-19 Direct Kit	260
DiaSorin MDx Simplexa Flu A/B and RSV Direct Kit	303
Hologic Aptima SARS-CoV-2	32
Hologic PANTHER Fusion SARS-CoV-2	6
Roche 6800 Influenza/COVID-19	36
Roche Liat Influenza/COVID-19	1
Luminex NxTAG RPP (v1)	114
Solana Respiratory Viral Panel	58
Quidel SARS	40
ThermoFisher Applied Biosystems TaqPath COVID-19	7

Across all targets, the NxTAG RPPv2 demonstrated high PPA (92.3%–100%) and NPA (99.5%–100%) relative to SoC testing (Table 6). At some participating sites, more than one SoC assay was used for comparison. Discrepant results were adjudicated by the NxTAG RPP with bidirectional sequencing.

**TABLE 6:** NxTAG RPPv2 performance comparison with the standard-of-care results

Target	Results per target	TP^*a*^	FP^*a*^	TN^*a*^	FN^*a*^	PPA^*a*^ (95% CI^*a*^) %	NPA^*a*^ (95% CI) %	Accuracy (95% CI) %
Adenovirus	973	46	2	923	2	95.8 (86–98.8)	99.8 (99.2–99.9)	99.6 (98.9–99.8)
*Chlamydia pneumoniae*	973	14	1	958	0	100 (78.5–100)	99.9 (99.4–100)	99.9 (99.4–100)
Coronavirus (HKU1, NL63, 229E, OC43)	973	65	1	905	2	97 (89.8–99.2)	99.9 (99.4–100)	99.7 (98.1–99.9)
Influenza A	2,044	122	3	1,919	0	100 (96.9–100)	99.8 (99.5–99.9)	99.9 (99.6–100)
Influenza B	2,044	41	0	2,003	0	100 (91.4–100)	100 (99.8–100)	100 (99.8–100)
Human metapneumovirus	1,031	39	1	990	1	97.5 (87.1–99.6)	99.9 (99.4–100)	99.8 (99.3–99.9)
*Mycoplasma pneumoniae*	973	48	0	921	4	92.3^*b*^ (81.8–97.0)	100 (99.6–100)	99.6 (98.9–99.8)
Parainfluenza 1	973	38	0	935	0	100 (90.8–100)	100 (99.6–100)	100 (99.6–100)
Parainfluenza 2	973	32	0	941	0	100 (89.3–100)	100 (99.6–100)	100 (99.6–100)
Parainfluenza 3	973	8	0	965	0	100 (67.6–100)	100 (99.6–100)	100 (99.6–100)
Parainfluenza 4	973	26	1	945	1	96.3 (81.7–99.3)	99.9 (99.4–100)	99.8 (99.3–99.9)
Rhinovirus/enterovirus	973	133	4	836	0	100 (97.2–100)	99.5 (98.8–99.8)	99.6 (98.9–99.8)
RSV A/B	1,904	77	4	1,823	0	100 (95.2–100)	99.8 (99.4–99.9)	99.8 (99.5–99.9)
SARS-CoV-2	1,818	234	5	1,576	3	98.7 (96.3–99.6)	99.7 (99.3–99.9)	99.6 (99.1–99.8)

^*a*^ TP, true positive; TN, true negative; FP, false positive; FN, false negative; PPA, positive percentage agreement; NPA, negative percentage agreement; CI, confidence interval.

^*b*^ The 52 positive *M. pneumoniae* results were obtained using BioFire RP methods.

### Reproducibility

A multicenter reproducibility study was conducted to evaluate the consistency of the NxTAG RPPv2 assay across testing sites, operators, days, and instruments. At each of three sites, two operators tested a nine-member reproducibility panel in four replicates over five non-consecutive days, yielding a total of 30 runs and 120 data points per site. The NxTAG RPPv2 assay demonstrated excellent reproducibility, with an overall percent agreement of 99.9% (Table S3).

## DISCUSSION

The NxTAG RPPv2 assay is an improved large-panel respiratory assay derived from the NxTAG RPP. The specific modifications implemented in RPPv2 compared with RPP are summarized in Table 7. No workflow differences exist between the two assays. A comparison of the LoD for each analyte on the NxTAG RPPv2 and the applicable comparator method is presented in Table 8. The LoD for SoC assays is not shown due to the number of different assays included in the comparison analysis.

**TABLE 7:** Modifications to the assay from NxTAG RPP to NxTAG RPPv2

Analyte	Change	Reason for change
Influenza A	Added primers	Improved coverage
	Removed primers	Strains no longer circulating
Influenza A H3	Added primers	Improved coverage
	Adjusted primer concentrations	Compensation for dropped primers
Human metapneumovirus	Added primers	Improved specificity
	Removed primers	Improved specificity
Rhinovirus/enterovirus	Adjusted primer concentrations	Improved specificity
SARS-CoV-2	New target	New pathogen of clinical importance

**TABLE 8:** Comparison of the limit-of-detection for each analyte between NxTAG RPPv2 and the applicable comparator methods

Analyte	Comparator method	NxTAG RPPv2 LoD	Comparator method LoD
Adenovirus	NxTAG RPP	2.01E+04 copies/mL (9.28E+01 TCID_50_/mL)	3.25E+00 TCID_50_/mL
*Chlamydophila pneumoniae*	NxTAG RPP	2.38E+02 copies/mL (3.74E+01 IFU/mL)	6.43E−02 TCID_50_/mL
Coronavirus 229E	NxTAG RPP	3.81E+02 copies/mL (1.22E−01 TCID_50_/mL)	1.07E−02 TCID_50_/mL
Coronavirus HKU1	NxTAG RPP	4.18E+03 copies/mL	2.15E+04 copies/mL
Coronavirus NL63	NxTAG RPP	1.00E+02 copies/mL (6.45E−03 TCID_50_/mL)	3.37E−03 TCID_50_/mL
Coronavirus OC43	NxTAG RPP	4.55E+03 copies/mL (7.32E−02 TCID_50_/mL)	7.15E−02 TCID_50_/mL
Human metapneumovirus	NxTAG RPP	2.62E+02 copies/mL (1.78E−02 TCID_50_/mL)	1.38E−01 TCID_50_/mL
Influenza A	NxTAG RPP	3.28E+02 copies/mL (3.74E−02 TCID_50_/mL)	5.53E−01 TCID_50_/mL
Influenza A 2009 H1N1	PCR/BDS	1.70E+03 copies/mL (1.12E−01 TCID_50_/mL)	3.28E+02 copies/mL
Influenza A H1	NxTAG RPP	1.60E+03 copies/mL (3.82E−01 TCID_50_/mL)	5.53E−01 TCID_50_/mL
Influenza A H3	NxTAG RPP	5.60E+01 copies/mL (2.15E−01 TCID_50_/mL)	9.36E−02 TCID_50_/mL
Influenza B	NxTAG RPP	6.33E+01 copies/mL (9.67E−01 TCID_50_/mL)	5.81E−01 TCID_50_/mL
*Mycoplasma pneumoniae*	NxTAG RPP	4.30E+03 copies/mL (5.56E+01 TCID_50_/mL)	1.42E+02 CCU/mL
Parainfluenza 1	NxTAG RPP	6.92E+02 copies/mL (7.64E−01 TCID_50_/mL)	2.82E+01 TCID_50_/mL
Parainfluenza 2	NxTAG RPP	3.45E+02 copies/mL (7.32E−01 TCID_50_/mL)	5.36E−01 TCID_50_/mL
Parainfluenza 3	NxTAG RPP	1.01E+03 copies/mL (1.10E+02 TCID_50_/mL)	1.61E+01 TCID_50_/mL
Parainfluenza 4	NxTAG RPP	1.69E+04 copies/mL (5.99E+01 TCID_50_/mL)	2.54E+00 TCID_50_/mL
RSV A	NxTAG RPP	4.97E+03 copies/mL (3.77E+01 PFU/mL)	2.15E+00 TCID_50_/mL
RSV B	NxTAG RPP	7.21E+03 copies/mL (3.20E−01 PFU/mL)	1.36E+00 TCID_50_/mL
Rhinovirus/enterovirus	NxTAG RPP	3.53E+03 copies/mL (2.30E+00 TCID_50_/mL)	3.34E+00 TCID_50_/mL
SARS-CoV-2	Simplexa COVID-19 Direct	5.00E+02 copies/mL (7.68E+00 TCID_50_/mL)	5.00E+02 copies/mL

The NxTAG RPPv2 assay demonstrated consistently high PPA and NPA across most viral and bacterial targets when compared to similar FDA-cleared assays, underscoring its reliability and clinical utility for multiplex respiratory pathogen detection. These findings are aligned with expectations for a high-throughput, syndromic molecular panel designed to streamline respiratory diagnostics in diverse clinical settings. Compared with a comparator method and bidirectional sequencing, the NxTAG RPPv2 achieved PPA values above 95% for 19 of the 20 viral targets, as well as for both bacterial targets. Notably, lower PPA values were observed for parainfluenza 4 virus (86.7%, 13/15) in the prospective arm where there was a low specimen size, but overall combined PPA was 90.3% (28/31).

The few instances of reduced PPA are likely multifactorial (Table 9). Contributing factors may include low pathogen load near the assay’s LoD, which can diminish sensitivity, particularly in frozen or archived specimens. Genetic variability in circulating strains may also affect primer and probe binding, especially for rapidly evolving RNA viruses such as influenza A (8). To further assess the potential impact of genetic diversity, *in silico* inclusivity analyses were performed against publicly available sequences for human coronavirus HKU1 and influenza A H1pdm09. This analysis demonstrated predicted inclusivity of approximately 99% for HKU1 sequences and 96% for influenza H1pdm09 hemagglutinin sequences, indicating broad coverage of currently circulating strains despite ongoing viral evolution (Table S4). Decreased nucleic acid extraction efficiency or degradation of unstable targets may also contribute to reduced detection, as can competitive inhibition during multiplex amplification, particularly when high-copy co-infecting organisms are present. With these potential limitations in mind, test results should not be used as the sole basis for diagnosis, treatment, or other patient management decisions; clinical presentation and additional testing should be considered to establish a final diagnosis for RTI. Despite these challenges, the NxTAG RPPv2 demonstrated robust overall performance across study sites and operators.

**TABLE 9:** Target limitations

Pathogen	Agreement issue	Evidence
Coronavirus HKU1	False negative for two specimens with confirmed coronavirus HKU1	Discordance linked to low viral loads and/or genetic diversity
Influenza A H1pdm09	Subtyping inconsistencies^*a*^	Primer mismatches historically, NxTAG RPPv2 improvements but may not cover all strains
Parainfluenza 4	Rarely detected	Limited specimen data affects assay validation and optimization
*M. pneumoniae*	Low bacterial load	NxTAG RPPv2 Limit of detection

^*a*^ Specimens with influenza A subtyping inconsistencies (or non-typeable) may be sent to the jurisdictional public health reference laboratories for further investigation.

Operational workflow is a critical determinant of diagnostic utility in clinical laboratories. The NxTAG RPPv2, in conjunction with eMAG/easyMAG extraction platforms, offers a high-throughput option not available with several competing systems. Although pre-analytical extraction requires specialized instrumentation, the downstream workflow is closed-tube, efficient, and requires less than 10 min of hands-on time for plate setup. While total hands-on time is somewhat greater than that of fully integrated systems (9), the ability to process up to 96 specimens within approximately 3 h after extraction provides substantial throughput advantages. In contrast, other multiplex respiratory panels may support smaller batch sizes, require longer turnaround times, or involve more manual handling steps. These operational features, combined with the economic feasibility of implementing the assay at scale, support its suitability for both routine laboratory use and surge testing during respiratory pathogen outbreaks.

The detection of multiple pathogens within a single specimen reflects the complex etiology of RTIs, particularly in vulnerable populations, such as children, the elderly, and immunocompromised patients. In this study, the observed co-infection rate of 11.3% among positive specimens aligns with previously reported frequencies, which range up to 48% depending on population, geography, and study design (10–13). Dual infections predominated, whereas triple or higher-order combinations were uncommon, a finding consistent with published surveillance data (10–13). From an epidemiological perspective, routine detection of co-infections provides valuable insight into pathogen interactions, their impact on disease severity, and their potential influence on outbreak dynamics and transmission patterns.

Beyond epidemiology, the rapid identification of respiratory pathogens using multiplex molecular assays has direct implications for patient management and public health. As novel antivirals emerge and molecular technologies advance, multiplex panels are expected to expand in scope, further improving diagnostic sensitivity and specificity. In clinical practice, rapid results facilitate timely infection control measures and antimicrobial stewardship by guiding appropriate isolation precautions and targeted therapy. By distinguishing bacterial from viral infections, multiplex testing can minimize unnecessary antibiotic exposure, reduce adverse drug effects, and mitigate antimicrobial resistance (14).

Looking ahead, the NxTAG RPPv2 assay is well-positioned to support outbreak preparedness and respiratory pathogen surveillance. Its broad target coverage, high throughput, and demonstrated reproducibility across laboratories make it suitable for both routine and surge testing. Integration of multiplex panels, such as the NxTAG RPPv2, into routine workflows enables real-time monitoring and differentiation of circulating respiratory pathogens and generation of actionable data for both patient care and public health response. The scalability and throughput of this assay, coupled with its cost-effectiveness, highlight its practical applicability during pandemics or seasonal surges. Furthermore, strong collaborations between clinical laboratories, public health agencies, and healthcare systems can amplify its impact, ensuring rapid dissemination of critical diagnostic information and optimizing resource utilization. As respiratory disease landscapes continue to evolve, flexible and reliable syndromic testing platforms will be increasingly critical for timely diagnosis, effective clinical management, and population-level surveillance.

### Conclusion

The NxTAG RPPv2 assay demonstrated high accuracy, reproducibility, and reliability for detecting viral and bacterial respiratory pathogens across diverse clinical settings. With its broad pathogen coverage, capability of differentiating RSV A and B, high-throughput workflow, and consistent performance in both fresh and frozen specimens, the panel provides a practical solution for routine diagnostic use. Its capacity to detect co-infections and deliver rapid, scalable results supports timely clinical decision-making, antimicrobial stewardship, and infection control efforts. Collectively, these features position the NxTAG RPPv2 as a valuable tool for enhancing patient care, strengthening respiratory pathogen surveillance, and improving outbreak preparedness.

## SUPPLEMENTAL MATERIAL

Supplemental tables jcm.01743-25-s0001.docx Tables S1 to S4.

ASM does not own the copyrights to Supplemental Material that may be linked to, or accessed through, an article. The authors have granted ASM a non-exclusive, world-wide license to publish the Supplemental Material files. Please contact the corresponding author directly for reuse.
