# Supplementary material for: High-throughput multiplex detection of respiratory pathogens: multicenter evaluation of the NxTAG Respiratory Pathogen Panel v2 assay
Source: J Clin Microbiol. 2026 Mar 19;64(4):e01743-25. doi: 10.1128/jcm.01743-25 (PMC13059723; doi:10.1128/jcm.01743-25)
Supplement: Supplemental tables — Tables S1 to S4. [file jcm.01743-25-s0001.docx]

**Supplemental Table 1. Targets on the NxTAG RPPv2 panel**

| **Viral Targets** | |
| --- | --- |
| Influenza A | Coronavirus NL63 |
| Influenza A – H1 | Coronavirus HKU1 |
| Influenza A – H1pdm09 | Human Metapneumovirus |
| Influenza A – H3 | Rhinovirus/Enterovirus |
| Influenza B | Adenovirus |
| Respiratory Syncytial Virus A | Parainfluenza virus 1 |
| Respiratory Syncytial Virus B | Parainfluenza virus 2 |
| SARS-CoV-2 | Parainfluenza virus 3 |
| Coronavirus 229E | Parainfluenza virus 4 |
| Coronavirus OC43 |  |
| **Bacterial Targets** | |
| *Chlamydia pneumoniae* | *Mycoplasma pneumoniae* |

**Supplemental Table 2. List of coinfection specimens detected by the NxTAG RPPv2.**

| **Analyte 1** | **Analyte 2** | **Analyte 3** | **# of specimens** | **NxTAG RPPv2 False Positives** | **NxTAG RPPv2 False Positive Analytes (#)** |
| --- | --- | --- | --- | --- | --- |
| Adenovirus | Coronavirus HKU1 |  | 1 | 0 | N/A* |
| Adenovirus | Coronavirus NL63 |  | 2 | 0 | N/A |
| Adenovirus | Coronavirus OC43 | Human Metapneumovirus | 1 | 1 | Human Metapneumovirus (1) |
| Adenovirus | Human Metapneumovirus |  | 4 | 1 | Adenovirus (1) |
| Adenovirus | Influenza A H3 |  | 1 | 1 | Influenza A H3 (1) |
| Adenovirus | Parainfluenza 2 | Human Metapneumovirus | 1 | 0 | N/A |
| Adenovirus | Parainfluenza 3 |  | 1 | 0 | N/A |
| Adenovirus | Parainfluenza 3 | Coronavirus NL63 | 1 | 0 | N/A |
| Adenovirus | RSV A |  | 1 | 0 | N/A |
| Adenovirus | Rhinovirus/Enterovirus |  | 18 | 2 | Adenovirus (2) |
| Adenovirus | SARS-CoV-2 |  | 1 | 0 | N/A |
| Coronavirus 229E | RSV A |  | 1 | 1 | Respiratory Syncytial Virus A (1) |
| Coronavirus 229E | Rhinovirus/Enterovirus |  | 2 | 0 | N/A |
| Coronavirus HKU1 | Human Metapneumovirus |  | 3 | 0 | N/A |
| Coronavirus NL63 | Coronavirus HKU1 |  | 1 | 0 | N/A |
| Coronavirus NL63 | Coronavirus OC43 |  | 1 | 0 | N/A |
| Coronavirus NL63 | Human Metapneumovirus |  | 3 | 0 | N/A |
| Coronavirus NL63 | Rhinovirus/Enterovirus |  | 6 | 0 | N/A |
| Coronavirus OC43 | Human Metapneumovirus |  | 1 | 0 | N/A |
| Coronavirus OC43 | Human Metapneumovirus | Rhinovirus/Enterovirus | 1 | 0 | N/A |
| Coronavirus OC43 | RSV B |  | 1 | 0 | N/A |
| Coronavirus OC43 | Rhinovirus/Enterovirus |  | 2 | 0 | N/A |
| Human Metapneumovirus | RSV A |  | 2 | 1 | Human Metapneumovirus (1) |
| Human Metapneumovirus | Rhinovirus/Enterovirus |  | 17 | 1 | Human Metapneumovirus (1) |
| Influenza A / Influenza A 2009 H1N1 | Coronavirus HKU1 |  | 1 | 0 | N/A |
| Influenza A / Influenza A 2009 H1N1 | Rhinovirus/Enterovirus |  | 5 | 0 | N/A |
| Influenza A / Influenza A H3 | Coronavirus 229E |  | 1 | 1 | Coronavirus 229E (1) |
| Influenza A / Influenza A H3 | RSV A |  | 1 | 1 | Respiratory Syncytial Virus A (1) |
| Influenza A / Influenza A H3 | Rhinovirus/Enterovirus |  | 3 | 0 | N/A |
| Influenza A / Influenza A H3 | SARS-CoV-2 | Rhinovirus/Enterovirus | 1 | 0 | N/A |
| Parainfluenza 1 | Rhinovirus/Enterovirus |  | 2 | 0 | N/A |
| Parainfluenza 2 | RSV A |  | 1 | 0 | N/A |
| Parainfluenza 3 | Coronavirus NL63 |  | 1 | 0 | N/A |
| Parainfluenza 3 | Coronavirus NL63 | Human Metapneumovirus | 1 | 1 | Human Metapneumovirus (1) |
| Parainfluenza 3 | Coronavirus OC43 |  | 2 | 0 | N/A |
| Parainfluenza 3 | Human Metapneumovirus |  | 1 | 0 | N/A |
| Parainfluenza 3 | RSV B |  | 1 | 0 | N/A |
| Parainfluenza 3 | Rhinovirus/Enterovirus |  | 3 | 1 | Parainfluenza 3 (1) |
| Parainfluenza 4 | Rhinovirus/Enterovirus |  | 5 | 2 | Parainfluenza 4 (2) |
| Parainfluenza 4 | SARS-CoV-2 |  | 1 | 1 | Parainfluenza 4 (1) |
| Parainfluenza 4 | SARS-CoV-2 | Rhinovirus/Enterovirus | 1 | 0 | N/A |
| Rhinovirus/Enterovirus | RSV A |  | 8 | 0 | N/A |
| Rhinovirus/Enterovirus | RSV B |  | 1 | 0 | N/A |
| SARS-CoV-2 | Coronavirus HKU1 |  | 1 | 0 | N/A |
| SARS-CoV-2 | Coronavirus OC43 |  | 4 | 1 | SARS-CoV-2 (1) |
| SARS-CoV-2 | Coronavirus OC43 | Rhinovirus/Enterovirus | 1 | 0 | N/A |
| SARS-CoV-2 | Human Metapneumovirus | Rhinovirus/Enterovirus | 1 | 0 | N/A |
| SARS-CoV-2 | RSV A |  | 1 | 1 | Respiratory Syncytial Virus A (1) |
| SARS-CoV-2 | Rhinovirus/Enterovirus |  | 4 | 2 | SARS-CoV-2 (2) |
| **Total** | | | **125** | **19** |  |
| **Total Double Infections** | | | **116** |  |  |
| **Total Triple Infections** | | | **9** |  |  |

*N/A: Not applicable

**Supplemental Table 3. NxTAG^®^ RPPv2 reproducibility**

| **Specimen** | **Target** | **Specimen Type** | **Agreement with Expected Results** | | | | |
| --- | --- | --- | --- | --- | --- | --- | --- |
|  |  |  | **Site 1** | **Site 2** | **Site 3** | **Overall** **(All Sites)** | |
| MA^a^1 | Influenza A H1pdm09 | Low Positive | 40/40  (100%) | 40/40 (100%) | 40/40  (100%) | 120/120 (100%) | 240/240 (100%) |
|  |  | Moderate Positive | 40/40  (100%) | 40/40 (100%) | 40/40  (100%) | 120/120 (100%) |  |
|  | Respiratory Syncytial Virus A | Low Positive | 40/40  (100%) | 40/40 (100%) | 40/40  (100%) | 120/120 (100%) | 240/240 (100%) |
|  |  | Moderate Positive | 40/40  (100%) | 40/40 (100%) | 40/40  (100%) | 120/120 (100%) |  |
|  | Rhinovirus^b^ | Low Positive | 40/40  (100%) | 40/40 (100%) | 40/40  (100%) | 120/120 (100%) | 240/240 (100%) |
|  |  | Moderate Positive | 40/40  (100%) | 40/40 (100%) | 40/40  (100%) | 120/120 (100%) |  |
|  | All other targets | Low Positive | 719/720 (99.86) | 720/720 (100%) | 720/720 (100%) | 2159/2160 (99.95%) | 4316/4320 (99.91%) |
|  |  | Moderate Positive | 718/720 (99.72%) | 720/720 (100%) | 719/720 (99.86%) | 2157/2160 (99.86%) |  |
| MA2 | Influenza A H3 | Low Positive | 39/40  (97.50%) | 40/40 (100%) | 40/40  (100%) | 119/120 (99.17%) | 239/240 (99.58%) |
|  |  | Moderate Positive | 40/40  (100%) | 40/40 (100%) | 40/40  (100%) | 120/120 (100%) |  |
|  | Respiratory Syncytial Virus B | Low Positive | 40/40  (100%) | 40/40 (100%) | 40/40  (100%) | 120/120 (100%) | 240/240 (100%) |
|  |  | Moderate Positive | 40/40  (100%) | 40/40 (100%) | 40/40  (100%) | 120/120 (100%) |  |
|  | All other targets | Low Positive | 717/720 (99.58%) | 720/720 (100%) | 720/720 (100%) | 2157/2160 (99.86%) | 4434/4440^c^ (99.86%) |
|  |  | Moderate Positive | 757/760 (99.61%) | 760/760 (100%) | 760/760 (100%) | 2277/2280 (99.87%) |  |
| MA3 | Influenza B | Low Positive | 39/40  (97.50%) | 40/40 (100%) | 40/40  (100%) | 119/120 (99.17%) | 239/240 (99.58%) |
|  |  | Moderate Positive | 40/40  (100%) | 40/40 (100%) | 40/40  (100%) | 120/120 (100%) |  |
|  | Parainfluenza virus 3 | Low Positive | 39/40  (97.50%) | 40/40 (100%) | 40/40  (100%) | 119/120 (99.17%) | 239/240 (99.58%) |
|  |  | Moderate Positive | 40/40  (100%) | 40/40 (100%) | 40/40  (100%) | 120/120 (100%) |  |
|  | *Mycoplasma pneumoniae* | Low Positive | 39/40  (97.50%) | 40/40 (100%) | 40/40  (100%) | 119/120 (99.17%) | 239/240 (99.58%) |
|  |  | Moderate Positive | 40/40  (100%) | 40/40 (100%) | 40/40  (100%) | 120/120 (100%) |  |
|  | All other targets | Low Positive | 718/720 (99.72%) | 720/720 (100%) | 720/720 (100%) | 2158/2160 (99.91%) | 4317/4320 (99.93%) |
|  |  | Moderate Positive | 719/720 (99.86%) | 720/720 (100%) | 720/720 (100%) | 2159/2160 (99.95%) |  |
| MA4 | SARS-CoV-2 | Low Positive | 40/40  (100%) | 40/40 (100%) | 40/40  (100%) | 120/120 (100%) | 240/240 (100%) |
|  |  | Moderate Positive | 40/40  (100%) | 40/40 (100%) | 40/40  (100%) | 120/120 (100%) |  |
|  | Human Metapneumo-virus | Low Positive | 40/40  (100%) | 40/40 (100%) | 40/40  (100%) | 120/120 (100%) | 240/240 (100%) |
|  |  | Moderate Positive | 40/40  (100%) | 40/40 (100%) | 40/40  (100%) | 120/120 (100%) |  |
|  | Adenovirus | Low Positive | 40/40  (100%) | 40/40 (100%) | 40/40  (100%) | 120/120 (100%) | 240/240 (100%) |
|  |  | Moderate Positive | 40/40  (100%) | 40/40 (100%) | 40/40  (100%) | 120/120 (100%) |  |
|  | All other targets | Low Positive | 716/720 (99.44%) | 720/720 (100%) | 720/720 (100%) | 2156/2160 (99.81%) | 4315/4320 (99.88%) |
|  |  | Moderate Positive | 719/720 (99.86%) | 720/720 (100%) | 720/720 (100%) | 2159/2160 (99.96%) |  |
| NEG | Negative | N/A | 840/840 (100%) | 840/840 (100%) | 840/840 (100%) | 2520/2520  (100%) | |
| ***Overall Agreement with Expected Results***  ***(all targets and all test levels)*** | | | **7499/7520 (99.72%)** | **7520/7520 (100%)** | **7519/7520**  **(99.99%)** | **22538/22560**  **(99.90%)** | |

^a^MA: multi analyte

^b^Reported by NxTAG RPPv2 as Rhinovirus/Enterovirus

^c^Excludes results of Influenza A matrix target at low positive concentration since the influenza A matrix LoD is 3-fold less sensitive than the subtype (H3). The influenza A matrix could not be assessed at 1.5× of the subtype LoD.

**Supplemental Table 4. Summary of *In Silico* Inclusivity Analysis Results for Coronavirus HKU1 and Influenza A H1**

| **Reportable Target** | **Inclusive Organism/Target** | **Total # Sequences in Alignment** | **# Sequences with Percent Oligo Identity ≥ 90%** | **Predicted Inclusivity Percentage (%)** † |
| --- | --- | --- | --- | --- |
| Coronavirus HKU1 ^a^ | Human coronavirus HKU1 | 480 | 477 | 99 |
| Influenza A H1 ^b^ | Influenza A pdm09 H1 (HA) | 45854 | 43835 | 96 |
| ^a^ Human coronavirus HKU1 sequences were retrieved from NCBI’s GenBank database.  All available sequences as of January 10, 2026 were assessed. | | | | |
| ^b^ Influenza A pdm09 H1 HA sequences retrieved from the GISAID EpiFlu database.  All human sequences collected from October 1, 2024 to January 12, 2026 were assessed. | | | | |
| † Rounded to the nearest whole number. | | | | |
